# Sex differences in the non-linear association between BMI and LDL cholesterol in type 2 diabetes

**DOI:** 10.3389/fendo.2023.1180012

**Published:** 2023-07-07

**Authors:** Kun Li, Bin Cao, Xiaojing Wang, Tao Chai, Jing Ke, Dong Zhao

**Affiliations:** ^1^Center for Endocrine Metabolism and Immune Diseases, Beijing Luhe Hospital, Capital Medical University, Beijing, China; ^2^Beijing Key Laboratory of Diabetes Research and Care, Capital Medical University, Beijing, China; ^3^Physical Examination Center, Beijing Luhe Hospital, Capital Medical University, Beijing, China

**Keywords:** BMI, LDL-C, type 2 diabetes mellitus, generalized additive models, sex differences

## Abstract

**Background:**

A data-based study reported the linear relationship between body mass index (BMI) and low-density lipoprotein cholesterol (LDL-C) in a normal population. However, there were no studies giving the suggestion for diabetes patients limited by sample size. This study aimed to investigate the non-linear dose-response relationship between BMI and LDL-C in type 2 diabetes mellitus (T2DM).

**Method:**

The study participants registered at the National Metabolic Management Center (MMC) of Beijing Luhe hospital from June 2017 to June 2021. T2DM was diagnosed according to the 1999 World Organization criteria. The generalized additive models (GAMs) were used to investigate the non-linear association between BMI and LDL-C. The relationship between BMI and LDL-C was visualized via the smooth splines function plot by sex. Segmented regressions were fitted to calculate the slopes with different estimated breakpoints.

**Results:**

After data cleaning, a total of 2500 participants with T2DM aged 30 to 70 years were included in this study. Compared with females, the spline between BMI and LDL-C showed an Inverted U shape in males. In males, the slopes below and above the breakpoint (26.08. 95% CI: 24.13 to 28.03) were 2.38 (95%CI: 1.06, 3.70) and -0.36 (95%CI: -1.20, 0.48), respectively.

**Conclusion:**

There was an Inverted U shape association between BMI and LDL-C in male participants with T2DM, for which the LDL-C was increased with BMI in the lean population, while LDL-C gradually tended to be flat or even decreased in the obese population. However, the Inverted U-shape between BMI and LDL-C was not found in female patients with T2DM.

## 1 Introduction

High body mass index (BMI) is a reliable obesity marker which has been used in numerous studies. A large cohort study suggests that high BMI is associated with the occurrence of cardiovascular diseases (CVD) (1). Overweight and obesity affect all individuals but are especially common among people with diabetes. In addition, patients with type 2 diabetes mellitus (T2DM) have a higher risk of cardiovascular disease (CVD) than the healthy population (2, 3).

It is well known that low level of high-density lipoprotein cholesterol (HDL-C) is a cause of cardiovascular disease, but recently the effect of low-density lipoprotein cholesterol (LDL-C) on CVD gradually be noticed (4–6). A cohort study has been reported that non-HDL cholesterol levels link to long-term risk of CVD (7). Moreover, among patients with obesity, LDL-C reduction with statins decreases CVD events, while drug treatments that improve atherogenic dyslipidemia have shown modest efficacy in CVD outcomes (8). Recent mendelian randomization studies also show that LDL-C is a risk factor for CVD (9, 10). These studies suggest that LDL-C concentration and its therapeutic modifications are also closely related to CVD, compared with HDL-C and TG (11).

A National Health and Nutrition Examination Survey (NHANEs) data-based study has been reported that the association between BMI and LDL-C in a normal population, the increase of LDL-C with BMI only in lean individuals, while LDL-C gradually tended to be flat or even decreased in obese people (12). Li’s study also gives a similar conclusion and found a non-linear relationship between BMI using Chinese adult data (13). However, due to sample size limitations, no study has made recommendations for patients with diabetes. In addition, a potential sex difference in the association between LDL-C and BMI should be illustrated in detail.

Therefore, this study aimed to investigate the association between BMI and LDL-C in Chinese adults with T2DM. We hypothesized that there was a non-linear dose-response relation between BMI and LDL-C in diabetes, and the sex difference existed. Our findings may identify the high CVD risk in T2DM to improve LDL-C and provide information on the prevention and treatment of CVD in Asian populations.

## 2 Methods

### 2.1 Study participants

This was a retrospective study. The participants with T2DM in this study registered at the National Metabolic Management Center (MMC) of Beijing Luhe hospital from June 2017 to June 2021. The participants registered at the National Metabolic Management Center (MMC), an innovation project for managing metabolic diseases and complications in China. The protocol of this project was published previously (14). In this study, participants were limited to 30-70 years when they accepted a visit.

Participants were excluded according to the following criteria: (1) pregnant or nursing women; (2) malignant tumor; (3) acute complications of diabetes; (4) used antihyperlipidemic agent.

The Medical Ethics Committee of Beijing Luhe Hospital, Capital Medical University approved the protocol. This study was performed by the Declaration of Helsinki, and all participants provided written informed consent.

### 2.2 Data collection

Data were collected by trained personnel according to the protocol. Blood samples were obtained in the morning after fasting for at least 8 hours. The questionnaire containing information on demographic characteristics, lifestyle factors (including alcohol drinking and cigarette smoking et al.), and medical history were administered by trained interviewers. For the participants who smoked daily or almost daily, smoking status was defined as ‘yes’. And for the participants who drank weekly or almost weekly, their drinking status was described as ‘yes.’ Education attainment was categorized as less than high school and high school or more. The history of hypertension was defined by a self-report or physician diagnosis.

Height and body weight were measured with a standard protocol at 8:00-10:00 AM after fasting at least 8 hours, and body mass index (BMI) was calculated as weight divided by height squared. Peripheral venous blood was withdrawn from all subjects in the morning after overnight fasting. Glycated hemoglobin (HbA1c) levels were assayed using high-performance liquid chromatography (HPLC) with a D10 set (Bio-Rad, Hercules, CA, USA). LDL-C, HDL-C, and triglyceride were measured using an auto-biochemical analyzer (Roche COBAS C501; Roche Diagnostics Corporation, Germany).

### 2.3 Definitions

T2DM was diagnosed according to the 1999 World Organization criteria if they had a fasting plasma glucose ≥ 7.0 mmol/L or 2-h plasma glucose ≥ 11.1 mmol/L or a self-reported physician diagnosis.

### 2.4 Statistical analysis

Data are presented as mean ± standard deviation (SD) or median [interquartile range (IQR)] values for continuous variables and as the frequency (%) for categorical variables. Data were tested for normal distribution and logarithmically transformed for statistical analysis when required. Differences between sex were assessed using the t-test or Mann-Whitney U test for continuous variables and Pearson’s χ^2^ test for categorical variables.

The generalized additive models (GAMs) were used to investigate the non-linear association between BMI and LDL-C (15). Models were adjusted for age, sex, education, smoking, drinking, course, hypertension, and HbA1c. The relationship between BMI and LDL-C was visualized using the smooth spline function plot by sex.

The segmented package of R was used to identify the breakpoints according to the generalized linear model. Therefore, two independent multivariable models were built before and after the breakpoints, which were adjusted for age, education, smoking, drinking, course, hypertension, and HbA1c. Multivariable models were constructed for males and females. In addition, the subgroups analysis was performed by age (30-<45, 45-<60, ≥60 years), and duration of diabetes (New-onset diabetes, course < 10 years, course ≥ 10 years).

R statistical software (version 4.1.2) with *mgcv, segmented, ggplot2*, and *stringr* packages were used for statistical analysis (16, 17). Two-tailed P <0.05 was considered statistically significant.

## 3 Results

### 3.1 Clinical characteristics

The data cleaning procedure can be seen in **Supplementary Figure 1**. After excluding participants using antilipemic agents and missing values of BMI and LDL-C, there were 2 500 participants aged 30-70 years including the current analysis. **Table 1** showed the demographic characteristics of the present study. The median (IQR) duration of diabetes was 58.00 (6.00, 131.00) months. There were significant differences between females and males regarding LDL-C, BMI, HDL-C, education level, drinking, smoking, and triglyceride (P <0.05).

**Table 1:** Demographic characteristics of the present study.

	Overall n=2500	Females n=1056	Males n=1444	P
Age, years, mean (SD)	51.04 (10.56)	53.59 (10.20)	49.09 (10.43)	<0.001
Course, months, median (IQR)	58.00 (6.00, 131.00)	70.00 (13.00, 145.00)	44.00 (2.00, 122.00)	<0.001
BMI, kg/m2, mean (SD)	26.56 (3.94)	26.35 (4.09)	26.72 (3.83)	0.020
LDL cholesterol, mg/dl, mean (SD)	124.08 (35.24)	126.84(35.03)	122.06 (35.26)	0.001
HbA1c, %, mean (SD)	8.99 (2.22)	8.98 (2.22)	8.99 (2.22)	0.911
HDL cholesterol, mg/dl, mean (SD)	46.15 (11.24)	49.40 (11.29)	43.78 (10.59)	<0.001
High school education, n (%)	1421 (57.0)	516 (49.00)	905 (62.80)	<0.001
Coronary atherosclerosis, n (%)	194 (7.8)	78 (7.4)	116 (8.0)	0.602
Hypertension, n (%)	894 (35.8)	420 (39.8)	474 (32.8)	0.001
Drinking, n (%)	1024 (41.0)	86(8.2)	938 (65.0)	<0.001
Smoking, n (%)	830 (33.2)	53 (5.0)	777 (53.8)	<0.001
Triglyceride, mmol/L, median (IQR)	1.52 (1.07, 2.36)	1.47 (1.06, 2.15)	1.56 (1.07, 2.51)	0.006
Fasting glucose, mmol/L, mean (SD)	9.57 (4.33)	9.29 (4.02)	9.77 (4.54)	0.006
HOMA-IR, mean (SD)	5.88 (10.42)	6.21 (7.89)	5.64 (11.95)	0.263

HbA1c, Glycated hemoglobin; BMI, body mass index; LDL, low-density lipoprotein; HDL, High-density lipoprotein; HOMA-IR, homeostasis model assessment of insulin resistance.

### 3.2 GAMs analysis

**Figure 1:**
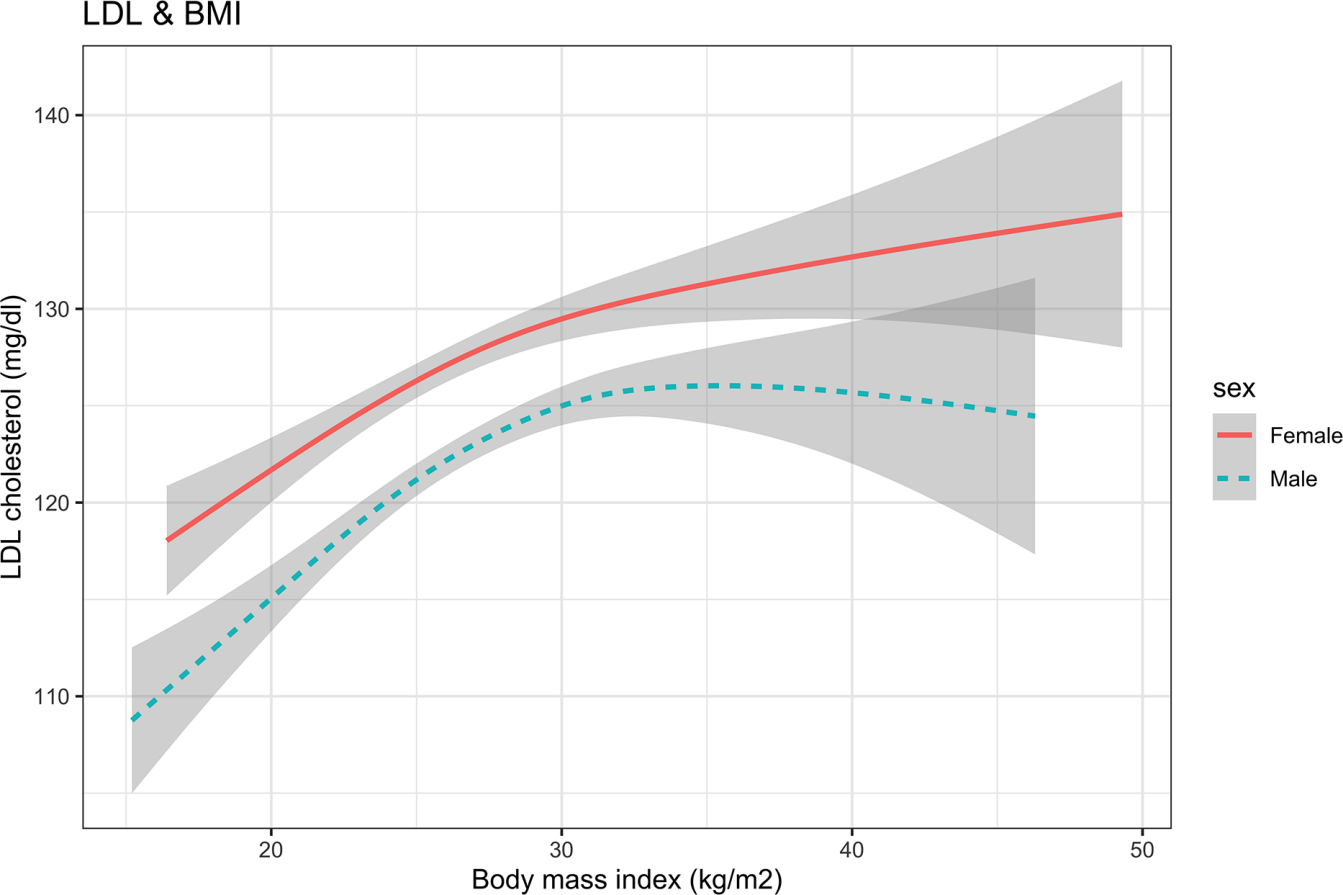
The nonlinear relationship between BMI and LDL cholesterol using a generalized additive model. BMI, body mass index; LDL, low-density lipoprotein.

The estimated regression coefficients of the generalized additive model are shown in **Table 2**. The intercept term, sex, education level, drinking, duration of diabetes, and HbA1c were significantly associated with LDL-C (P<0.05). **Figure 1** shows the non-linear association between BMI and LDL-C in females and males. The spline curve of LDL in females presents a continuously increased trend with BMI increasing. There was a breakpoint, and the spline curve increased gradually after the breakpoint. However, compared with females, the spline curve of males shows an Inverted U shape.

**Table 2:** Estimated regression coefficients (β) from the generalized additive model.

	β (SE)	P value
Intercept	105.08 (5.53)	<0.001
Sex		
Female	Reference	
Male	-12.43 (1.82)	<0.001
Age		
Per 1 year	-0.10 (0.08)	0.221
Education		
Below high school	Reference	
High school or above	7.18 (1.48)	<0.001
Smoking		
No	Reference	
Yes	2.09 (1.73)	0.225
Drinking		
No	Reference	
Yes	7.28 (1.73)	<0.001
Course	-0.03 (0.008)	<0.001
Hypertension		
No	Reference	
Yes	-1.08 (1.51)	0.483
HbA1c	2.93(0.31)	<0.001

HbA1c, Glycated hemoglobin.

The results of the threshold analysis were shown in **Table 3**. For females, the breakpoint was 27.20 (95%CI: 24.35, 30.05), BMI was linearly associated with LDL-C below the breakpoints (slope: 4.37, 95%CI: 1.24 to 7.50), and a gradual increase above the breakpoint (slope:0.05, 95%CI: -0.64 to 0.73, P <0.001). Compared with the results of females, the curve of LDL increased first and then decreased after the breakpoint. In the results of males, the slopes below and above the breakpoint (26.08. 95% CI: 24.13 to 28.03) were 2.38 (95%CI:1.06, 3.70) and -0.36 (95%CI: -1.20, 0.48), respectively.

**Table 3:** The estimated beak points and slope between BMI and LDL-C.

Population	BMI(kg/m2) Estimated Breakpoint (95%CI)	LDL cholesterol slope (mg/dl per kg/m2)		P value for differences
		<Breakpoint (95% CI)	>=Breakpoint (95% CI)	
Female				
Model 1	27.40(24.55,30.25)	3.78(0.99,6.56)	0.11(-0.61,0.82)	<0.001
Model 2	27.20(24.35,30.05)	4.37(1.24,7.50)	0.05(-0.64,0.73)	<0.001
Male				
Model 1	26.08(23.85,28.31)	2.18(0.88,3.49)	-0.40(-1.24,0.45)	<0.001
Model 2	26.08(24.13,28.03)	2.38(1.06,3.70)	-0.36(-1.20,0.48)	<0.001

Model 1: Adjusted for age, duration of diabetes, HbA1c, smoking, drinking and education level. Model 2: Adjusted for covariates in model 1 plus hypertension and Coronary atherosclerosis. BMI, body mass index; LDL, low-density lipoprotein.

### 3.3 Subgroup analysis

**Figure 2:**
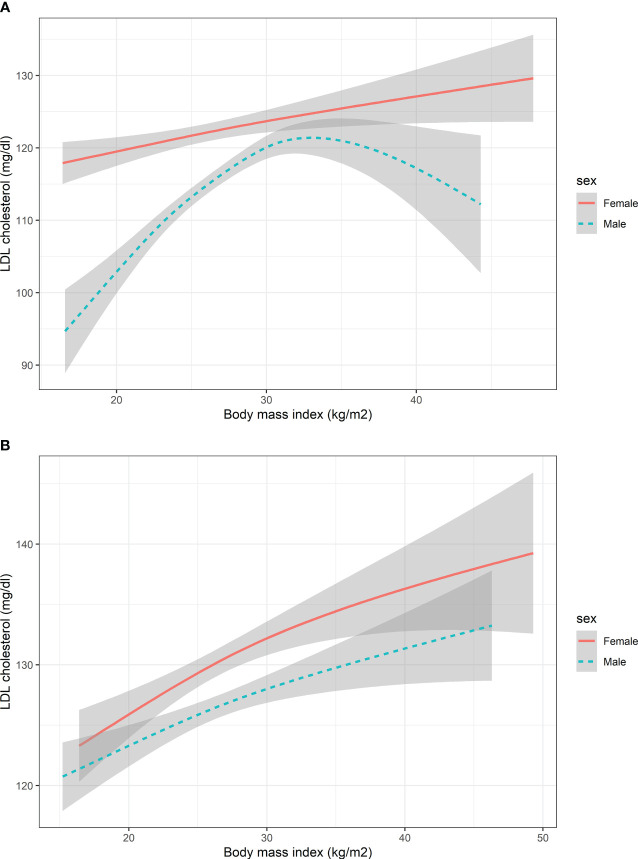
The nonlinear relationship between BMI and LDL cholesterol with subgroups of HbA1c. **(A)** Participants with HbA1c<9% **(B)** Participants with HbA1c>=9%. HbA1c, Glycated hemoglobin; BMI, body mass index; LDL, low-density lipoprotein. The number of participants in the group of HbA1c<9% and HbA1c>=9% was 1353 and 1147, respectively.

The non-linear association between BMI and LDL-C for HbA1c groups in males and females was shown in **Figure 2**. The spline did not show an Inverted U shape in males with HbA1c > 9% compared with the group of HbA1c ≤9%. The shape of the splines was similar in the females’ subgroup of HbA1c > 9% and HbA1c ≤9%.

**Figure 3:**
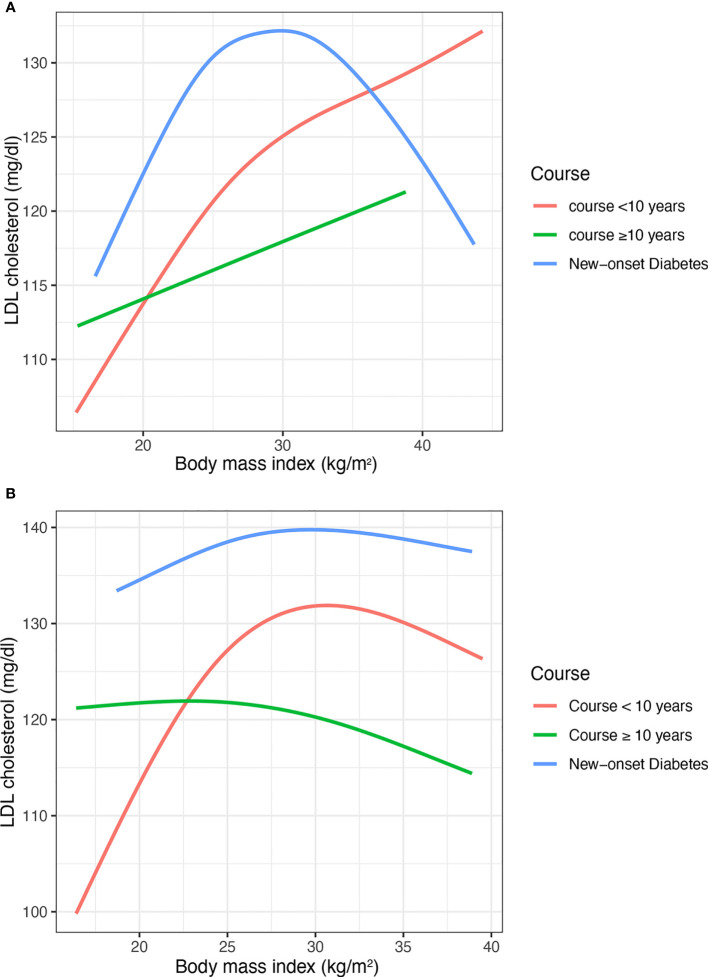
The nonlinear relationship between BMI and LDL cholesterol with subgroups of the duration of diabetes. **(A)** Males **(B)** Females. BMI, body mass index; LDL, low-density lipoprotein. The number of participants in the group of new-onset diabetes, course<10 years, course>=10 years and was 460, 1281, and 759, respectively.

**Figure 4:**
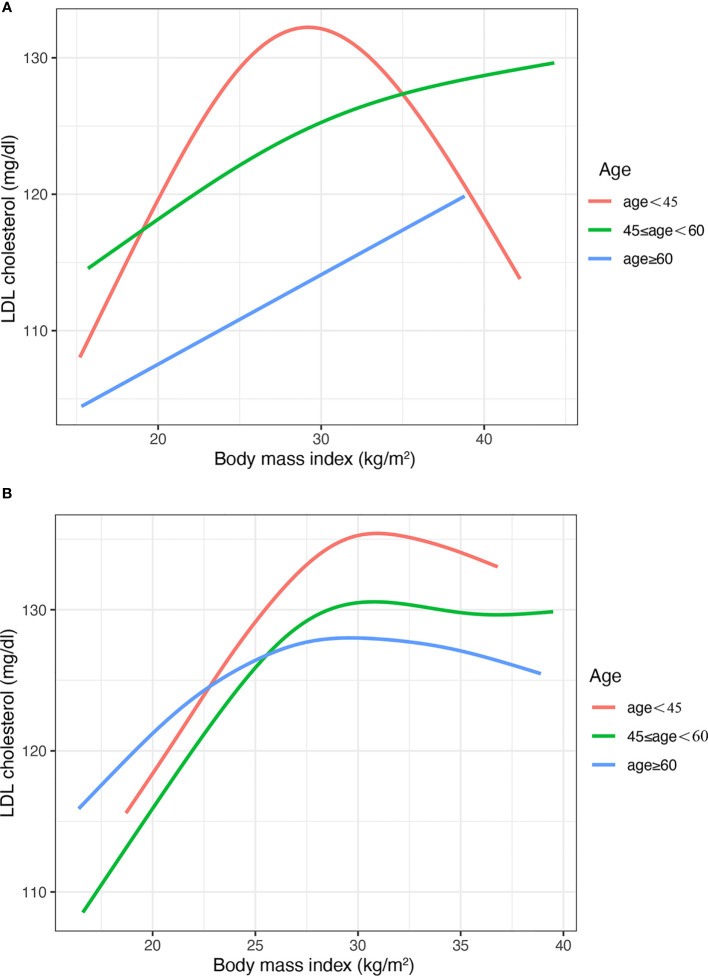
The nonlinear relationship between BMI and LDL cholesterol with subgroups of age. **(A)** Males **(B)** Females. BMI, body mass index; LDL, low-density lipoprotein. The number of participants in the group of age <45 years, 45-<60 years and >60 was 472, 610, and 1418, respectively.

In the subgroup of course (**Figure 3**) and age (**Figure 4**), the Inverted U shape of the spline could be seen in the new-onset diabetes groups and age <45 years group of males, respectively.

## 4 Discussion

In this study, we found an Inverted U-shaped association between BMI and LDL-C in male patients with T2DM, for which the LDL was increased with BMI in the relatively lean population, while LDL-C gradually tended to be flat or even decreased in the more obese population. However, the inverted U-shaped smooth curve of BMI and LDL-C was not found in female patients with T2DM. Similar results have been reported in the normal population (13, 18). However, there were no studies giving the suggestion for diabetes patients limited by sample size.

Moreover, the spline did not show an Inverted U shape in males with HbA1c > 9% compared with the group of HbA1c ≤9%, which means that the sex difference of association between LDL and BMI vanished in the population with poor HbA1c control. In fact, HbA1c is directly correlated with higher LDL, especially in people with poor blood glucose control (**Supplement Figure 2**). This confounding effect resulted in the disappearance of gender differences in the present study. In the future, more mechanistic studies are needed to elucidate this phenomenon.

To our knowledge, this was the first study aimed to investigate the non-linear dose-response relationship between BMI and LDL-C in diabetes, which may help to identify the high CVD risk of diabetes patients. Assessing the association between BMI and CVD risk factors can help to prevent the onset or progression of the disease and explaining the non-linear relationship between the two can help quantify the risk levels of people with different BMI levels (13, 19, 20).

The association between BMI and lipid metabolism was estimated by TG and HDL-C in the previous studies. However, there was insufficient evidence of the effect on LDL-C (21). The phenomenon of the sharp truncation between BMI and LDL-C was previously overlooked. The mechanisms of this situation may be explained by the changes in the efficiency of lipid deposition in the adipose tissue for obesity (22). In the lean population, normal fat production implies that lipoprotein lipase rapidly offloads fatty acids from triglyceride-rich fats, converting them to cholesterol-rich LDL-C (23). Therefore, weight gain is associated with an increase in these lipid components. However, when lipid deposition efficiency in adipose tissue is reduced due to reaching functional limits, triglycerides may be stagnated in VLDL (13, 24). This was consistent with the results in the present study that LDL-C remained stable or decreased with increasing BMI in the obese population.

There are some differences in the results between the normal population and diabetes patients. Compared with the second slope (>=Breakpoint) of the model between BMI and LDL-C in normal males, the slope of the model in diabetes of males was smoother (**Table 3**). Moreover, the value of the breakpoint of the model between BMI and LDL-C in diabetes of males was larger than the value in normal males. The reason for this may be that the average BMI of the participants in this study was higher than the normal population.

In addition, the results were similar for the age and course subgroups. The similar trend of the curve could be explained by the fact that patients with a shorter course of diabetes are younger. The Inverted U shape was more pronounced in the subgroup with a shorter duration of diabetes and younger age. However, the Inverted U shape was not found in these people, who were elder and had a long course of diabetes. Li’s studies also found the age difference in the association between BMI and LDL-C, which was consistent with the results of this study (13).

The sex difference in the association between BMI and LDL may be explained by the following reason. First, sex-specific hormones could lead to sex differences in lipid metabolism, the previous studies suggested that the level of LDL-C was associated with hormones (25, 26). The level of LDL-C is correlated with menopausal status, that is, postmenopausal females have higher concentrations of LDL-C (27). In addition, a previous study suggested that hormone replacement therapy could modify the activity of LDL receptors (28). Thus, differences in hormonal status between males and females may lead to different associations between BMI and LDL-C. Second, there are sex differences in the concentrations of some cholesterol-regulating substances, which may affect lipid metabolism. For example, there was a higher PCSK9 concentration in females than in males (29). Third, sex-specific BMI and LDL-C associations may be influenced by differences in the distribution of adipose tissue between males and females. Females store more lipids and have higher percent body fat, less visceral white adipose tissue, and more subcutaneous adipose tissue than males (30). Moreover, females have a higher rate of TG synthesis compared to males. More mechanistic studies should be done to support this point.

Although a previous study reported the linear association linear relationship between BMI and LDL-C in a normal population (30), we made some substantial contributions to the literature. First, the primary results were analyzed with a high degree of accuracy using data from a national project to manage metabolic patients and according to the same standard. Second, we addressed sex differences in the non-linear association between BMI and LDL-C using generalized additive models in diabetes patients, which could explain a detailed quantification of ascending and descending slopes through dose-response analyses. Finally, in the context of the current epidemic of diabetes, the focus of this study on diabetes has important implications for the prevention and treatment of diabetes.

This study also has several limitations. First, we can’t infer causality and must highlight reverse causality as a possibility due to the cross-sectional study design. More cohort studies should be designed to clarify the association between BMI and LDL-C in type 2 diabetes. In future research, we will analyze the association between BMI and the index of lipid metabolism in a cohort using causal inference methods. Second, we could not get the indirect obesity index (e.g., body fat percentage); it was not analyzed in the present study. Third, although the result of the association between BMI and LDL-C was adjusted by a series of sensitivity analyses, residual confounders still existed, such as the usage of hormones and other drugs. Fourth, the GAM method aims to describe the nonlinear trend between two variables. It illustrates the trend in graphs, so we can use a smaller sample size for modeling. Although the GAM method does not suggest a minimum sample for modeling, a larger sample size may make the result more powerful (31). It’s difficult to get more data on diabetes from a single center. In the future study, we will include multi-center data and expand the sample to verify the conclusions of this study. Last, we could not include the patients with type 1 diabetes mellitus who have younger ages and morbid state. More studies are needed to explore the relationship between LDL-C and BMI in type 1 diabetes.

## 5 Conclusion

There was an Inverted U shape association between BMI and LDL-C in male patients with T2DM, for which the LDL-C was increased with BMI in the lean population, while LDL-C gradually tended to be flat or even decreased in the obese population. However, the Inverted U-shaped smooth curve of BMI and LDL-C was not found in female patients with T2DM.

## Data availability statement

The data analyzed in this study is subject to the following licenses/restrictions: The raw data supporting the conclusions of this article will be made available by the authors, without undue reservation. Requests to access these datasets should be directed to Zhao Dong, zhaodong@ccmu.edu.cn.

## Ethics statement

The studies involving human participants were reviewed and approved by Medical Ethics Committee of Beijing Luhe Hospital, Capital Medical University. The patients/participants provided their written informed consent to participate in this study.

## Author contributions

LK: Conceptualization, methodology, analysis, writing- original draft and editing. CB and WX: Methodology, writing-review and editing. KJ and CT: Methodology. DZ: Conceptualization, methodology, writing- review and editing, supervision. All authors contributed to the article and approved the submitted version.
