# Supplementary material for: Sex differences in the non-linear association between BMI and LDL cholesterol in type 2 diabetes
Source: Front Endocrinol (Lausanne). 2023 Jul 7;14:1180012. doi: 10.3389/fendo.2023.1180012 (PMC10360932; doi:10.3389/fendo.2023.1180012)
Supplement: Supplementary file 1 [file Image_1.pdf]

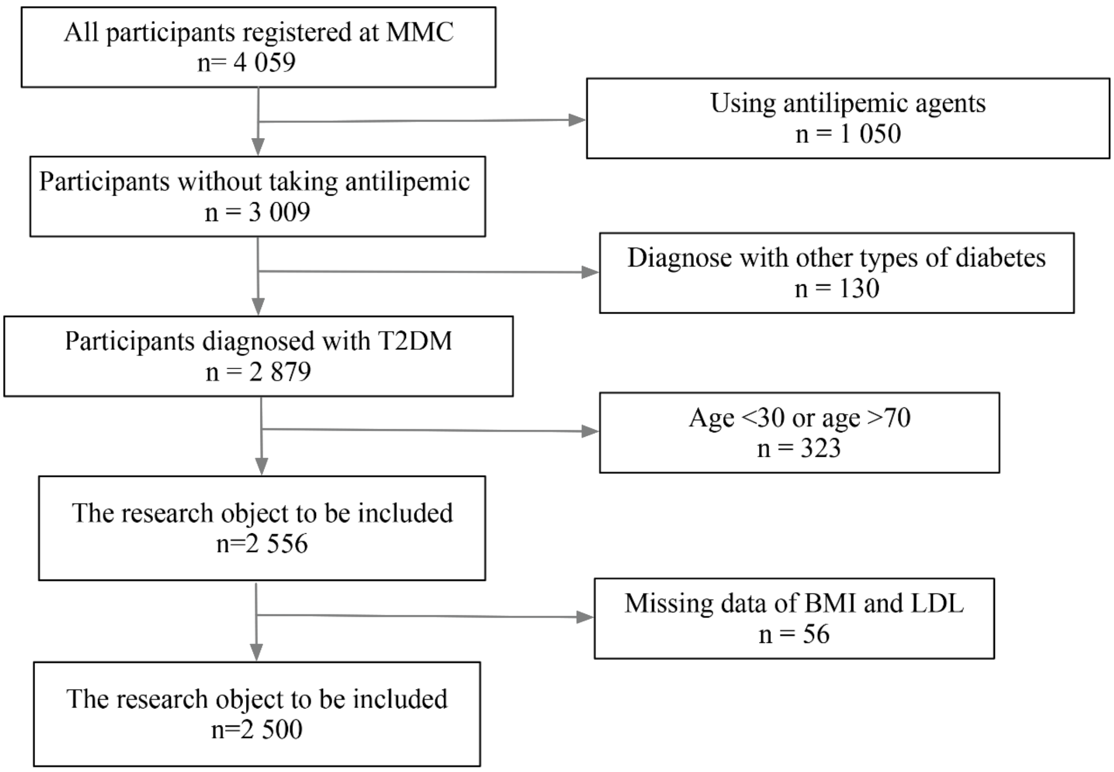

Supplemental figure 1. Data cleaning procedure of the present study.
MMC, National Metabolic Management Centers; T2DM, type 2 diabetes mellitus.

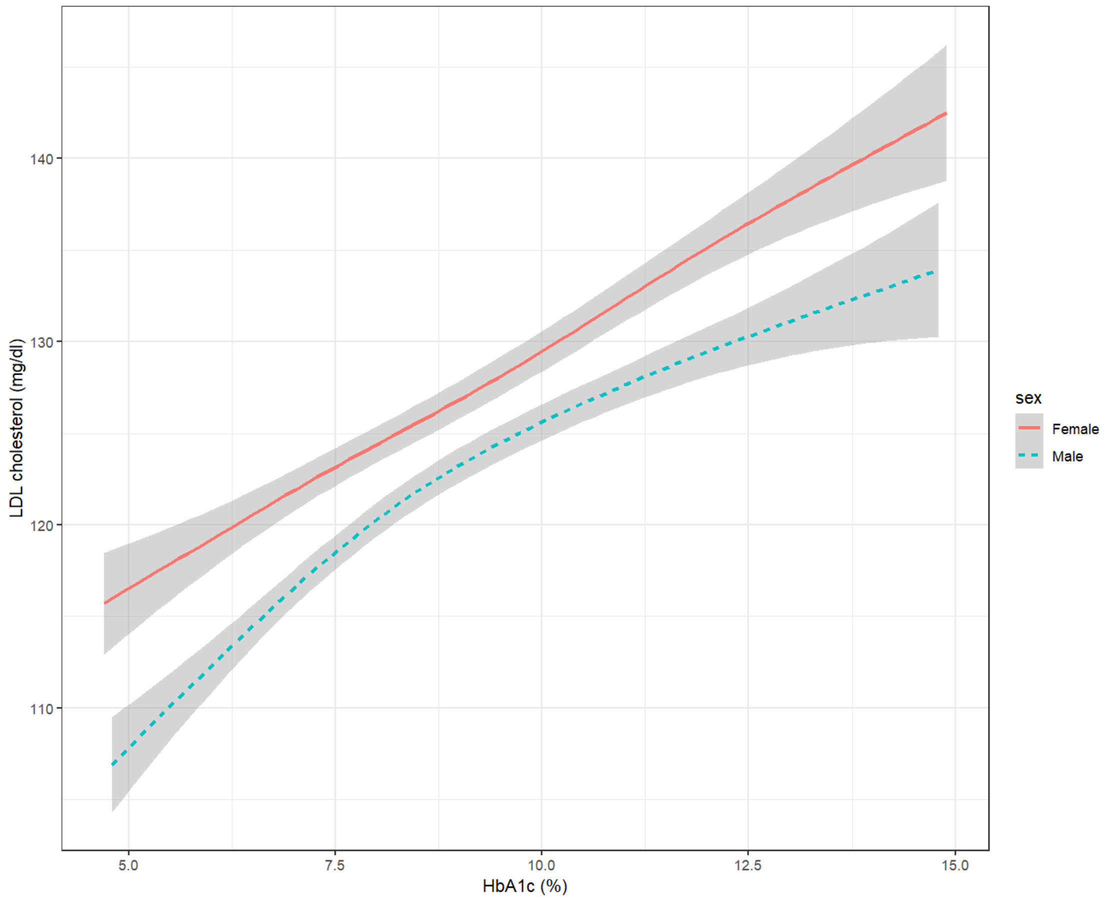

Supplemental figure 2. LDL cholesterol was associated with HbA1c in type 2 diabetes patients. Glycated hemoglobin, HbA1c.
